# The significance of p40 expression in sclerosing hemangioma of lung

**DOI:** 10.1038/srep06102

**Published:** 2014-08-18

**Authors:** Jian Wu, Chang Zhang, Haiguo Qiao

**Affiliations:** 1 Department of pathology, Huaian First People's hospital, 6 Beijing Road West, Jiangsu 223300, China; 2 Department of Pathology, Lianshui County hospital, Jiangsu, 223400, China

## Abstract

To explore the histogenesis of cuboidal and polygonal tumor cells in the sclerosing hemangioma of lung (SHL), eighteen cases of SHL were retrospectively studied. SPB, p40, TTF-1,EMA,CKpan, vimentin,SMA, CgA,Syn and CD34 were immunohistochemically labeled by the EnVisionmethod. It was found that the four main types of structure in SHL were solid,papillary, hemorrhagic and sclerotic patterns. The tumor cells were composed mainly of two types of cells: cuboidal tumor cells and polygonal tumor cells. The immunohistochemistry showed that p40 was expressed only in cuboidal tumor cells. TTF-1 and EMA were expressed in both polygonal cells and cuboidal cells. SPB was also expressed in cuboidal tumor cells; vimentin was expressed in all polygonal tumor cells and some cuboidal cells. The findings suggest that the p40-positive cuboidal tumor cells may be pluripotent original respiratory epithelial cells, with multi-directional differentiation capacity.

The sclerosing hemangioma of lung (SHL) is an uncommon and uncertain histogenetic benign tumor first described by Liebow and Hubell in1956^1^. The histopathological morphology of SHL has been studied extensively by many investigators^2^,^3^,^4^,^5^,^6^,^7^,^8^,^9^. SHL is a well-circumscribed parenchymal tumor, which consists of two types of tumor cells: cuboidal tumor cells and polygonal tumor cells. In SHL, there are mainly four types of structure: solid, papillary, hemorrhagic and sclerotic patterns. However, the histogenesis of SHL has remained controversial. Previously, the cuboidal cells were considered to be entrapped alveolar pneumocytes and bronchiolar epithelium, and polygonal cells were considered to be tumor cells. Now, both cuboidal cells and polygonal cells are considered to be tumor cells present in the same tumor^10^. The p40 antibody (antibody recognizing ΔNp63 only)isanisoform of p63. The single expression of ΔNp63 is seen in stem-like cell populations and may contribute significantly to biological processes such as proliferation and differentiation of epithelia. The objective of this study is to explore the histogenesis of SHL by analyzing the clinical pathological data and p40 immunohistochemical results of 18 cases of SHL.

## Results

### The clinical data

Among the 18 cases of SHL, there were 16 women and 2 men, indicating a female/male ratio of 8:1. The patients ranged in age from 26 years to 58 years old, with a mean age of 44.6 years. The course of disease varied from 20 days to 2 years. All patients experienced cough, sputum production, chest pain, chest tightness, and chest discomfort. Ten tumors occurred in the right lower lobes, four in the left lower lobes, one in the right upper lobes, three in the left upper lobes (Table 1). The radiological appearance indicated a solitary mass or nodule in all cases.

### Pathology observation

**Figure 1:**
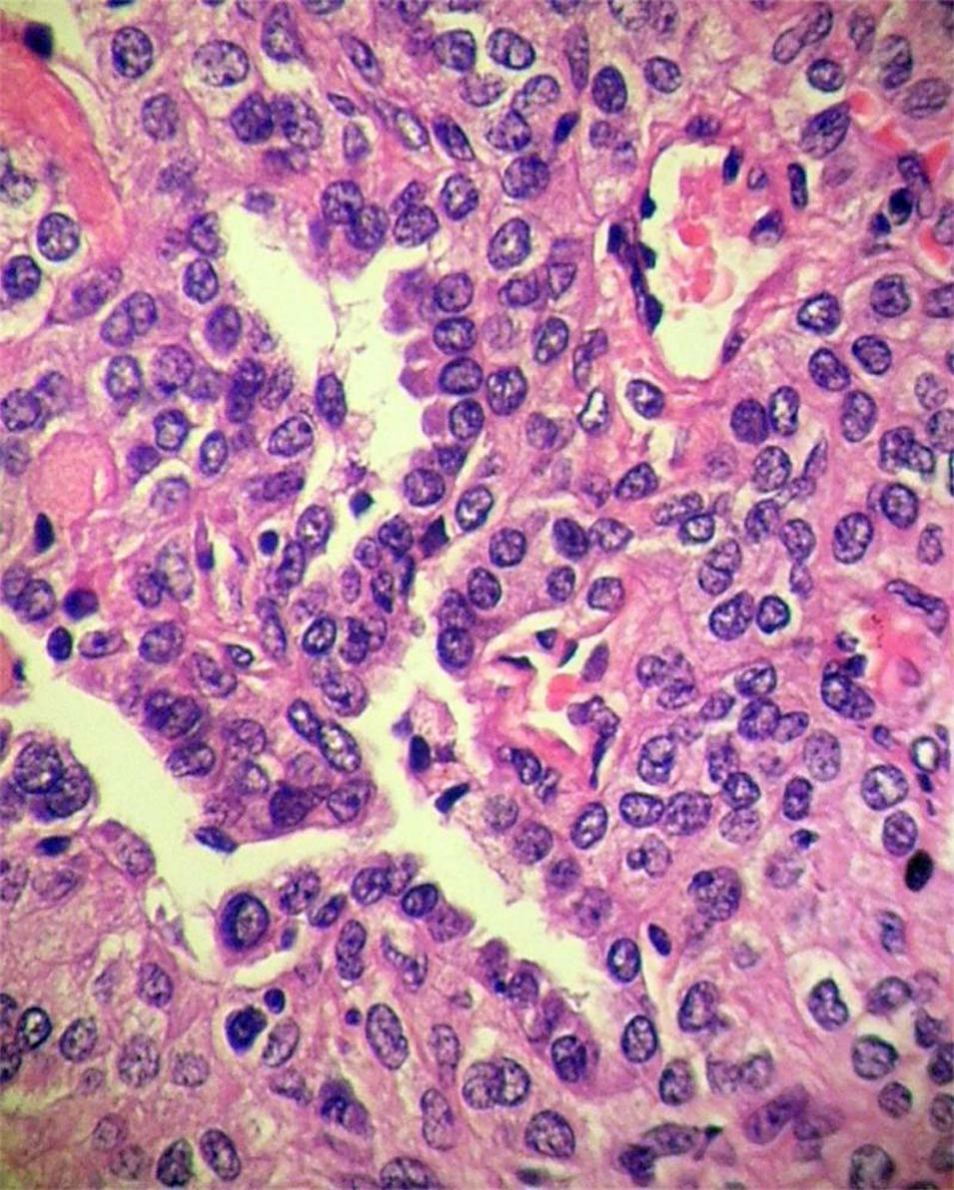
There are two kinds tumor cells in SHL: cuboidal tumor cells and polygonal tumor cells. HE × 400.

Gross features - A solitary tumor was observed in the parenchyma in part of the lung tissue removed by surgery, with a well-circumscribed mass or nodule that was easy to separate from surrounding pulmonary tissues. The mass or nodule was round or oval,with a diameter of 1 cm to 4 cm (average diameter of 3 cm). The tumors' cut surfaces presented as meaty, coloring from gray-tan to darkred. Histologic features - All 18 cases showed the typical histologic appearances of SHL described below: there were two types of tumor cells (Figure 1): cuboidal tumor cells and polygonal tumor cells. Polygonal tumor cells showed uniform, medium-size polygonal or round nuclei with moderate amounts of eosinophilic or clear cytoplasm. Cuboidal tumor cells were characterized by a small and deeply stained nucleus. Neither necrosisn or mitotic activity was found in these two types of tumor cells in SHL. Tumor structure showed solid, papillary, hemorrhagic and sclerotic patterns. In the solid area, the aggregation of polygonal cells was observed. The cuboidal tumor cells formed glandular-like structures or clefts. In the papillary area, the papillary surface was lined with cuboidal tumor cells. The polygonal tumor cells were visible in the papillary axis. The sclerotic area was formed by hyaline collagen tissues and the proliferation of fibrous tissue. Hemorrhagic areas were filled with vascular-like channels or true vessels which were packed with varying amounts of erythrocytes. Typical polygonal tumor cells were always in the interstitium. Lymphocytes, hemosiderin, cholesterol fissures, xanthomacells and calcification could be observed in interstitium.

### Immunohistochemistry

**Figure 2:**
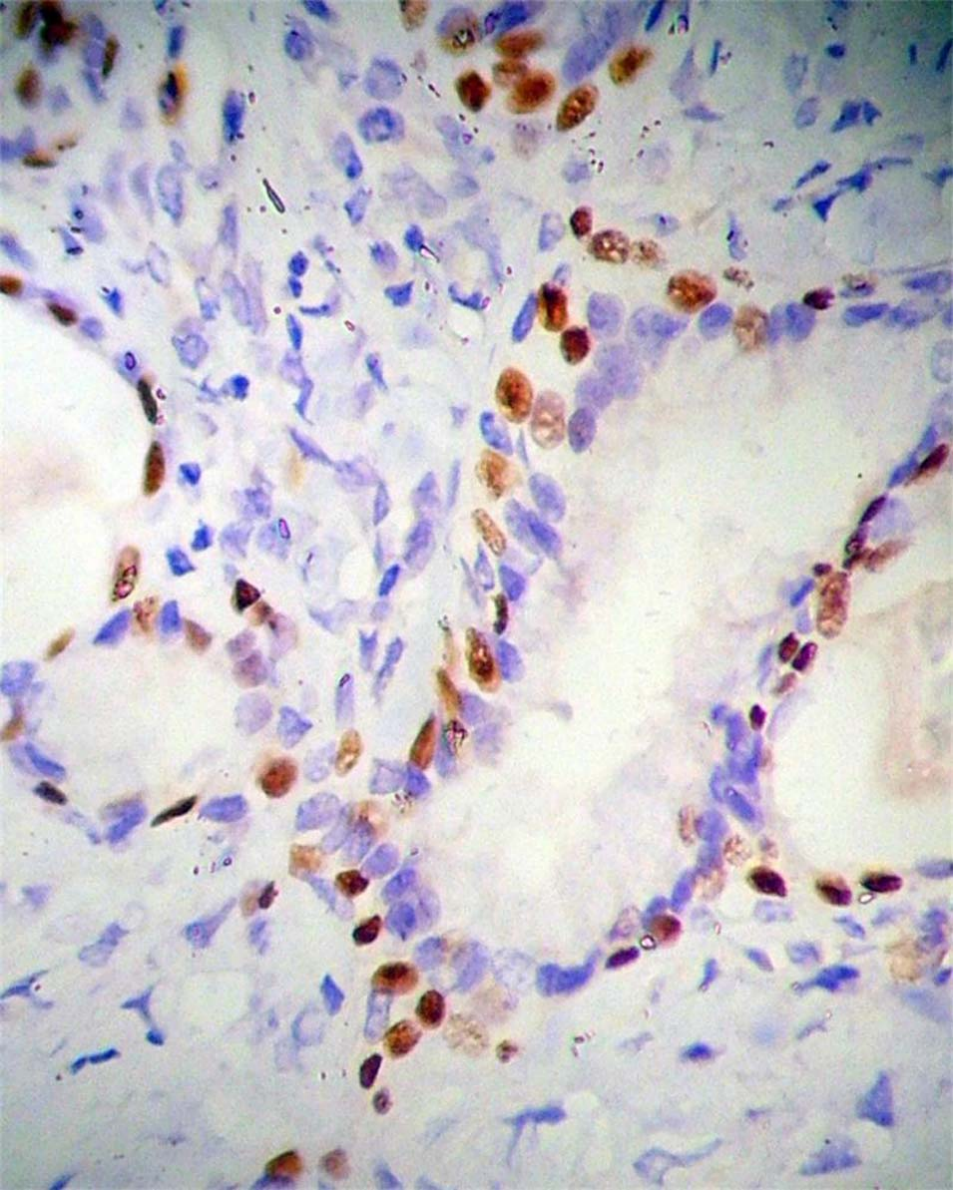
P40 positive expression in cuboidal tumor cells and no positive expression in polygonal tumor cells in SHL. EnVision method × 400.

**Figure 3:**
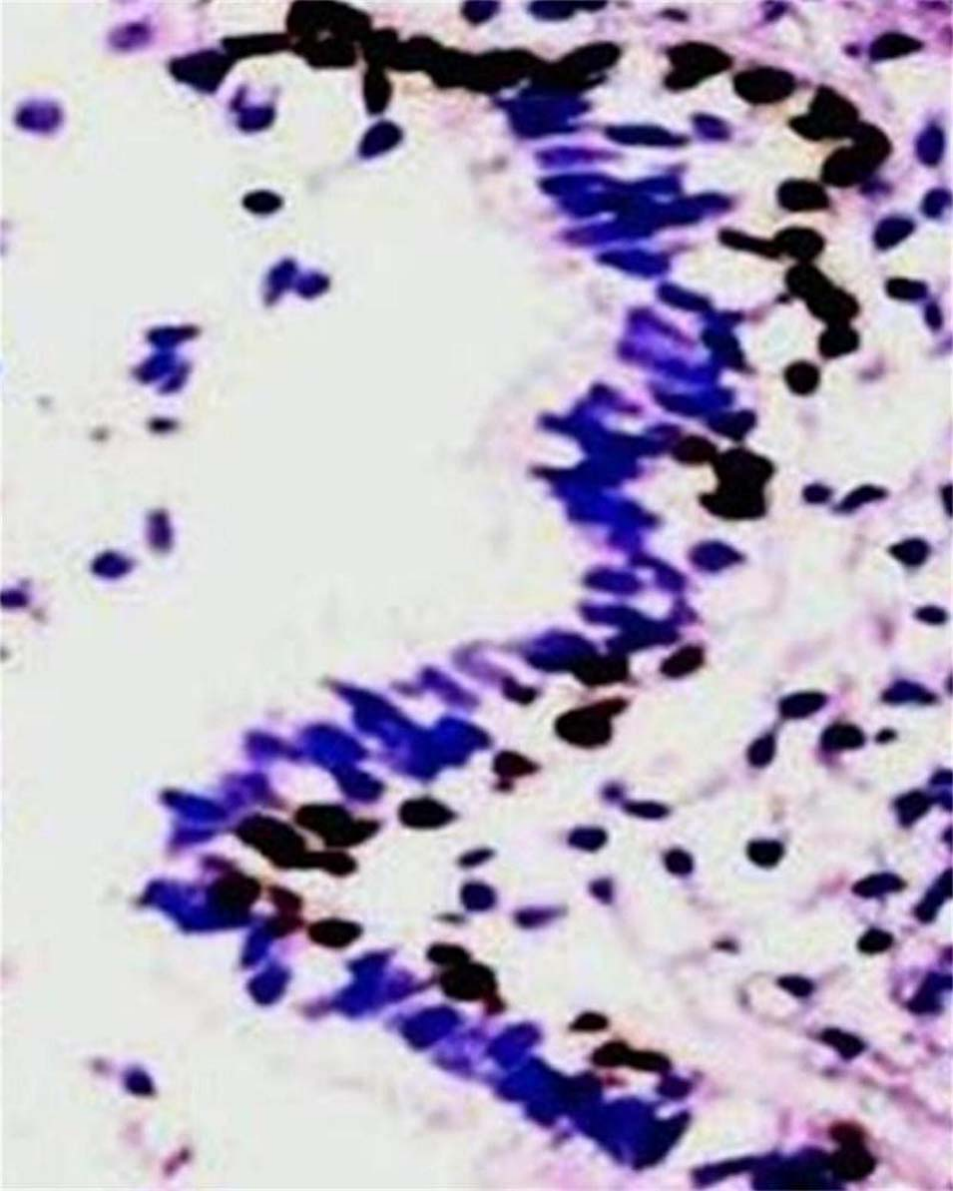
P40 positive expression in basal cells of glandular structures of bronchi in SHL. EnVision method × 400.

**Figure 4:**
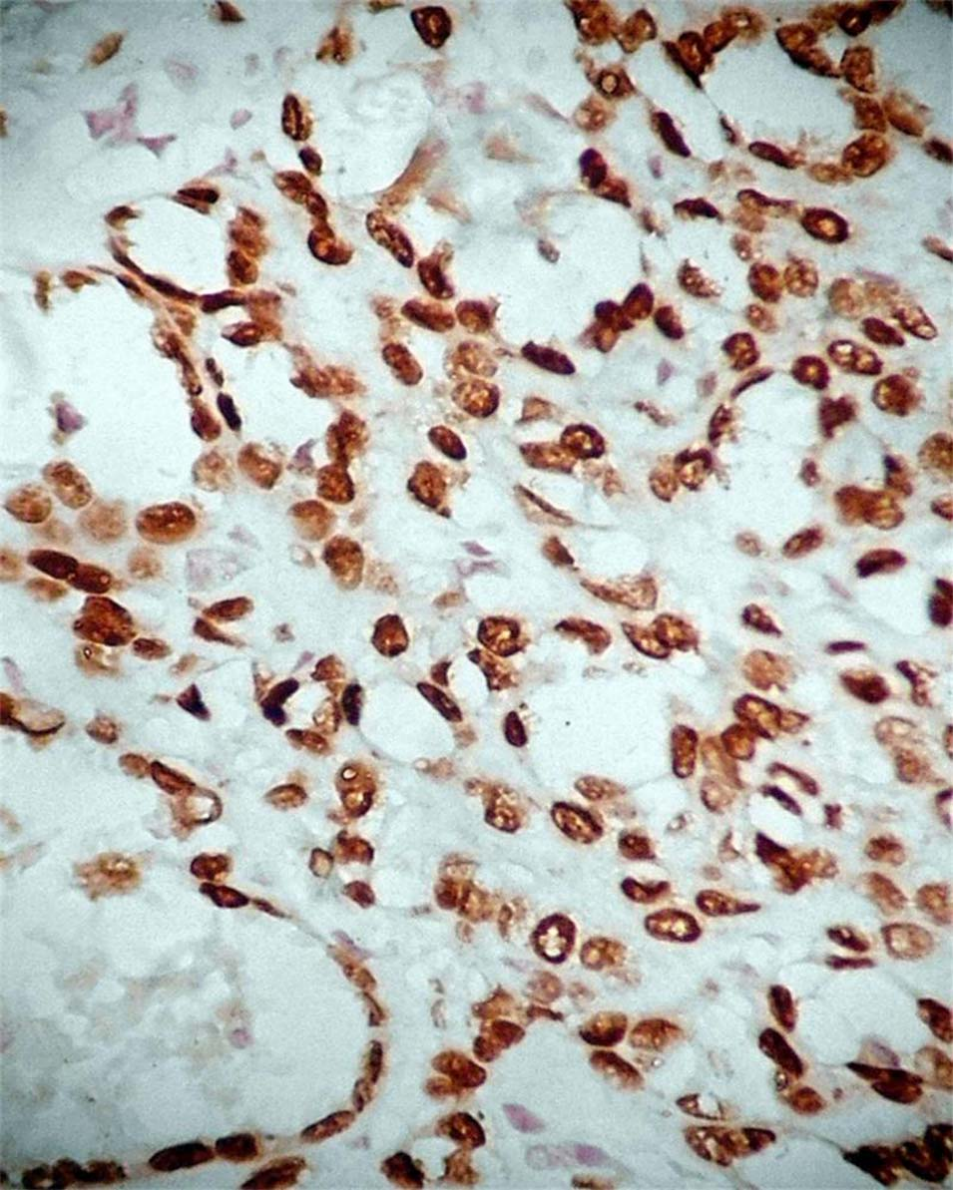
TTF-1 positive expression in both the cuboidal tumor cells and polygonal tumor cells in SHL. EnVision method × 400.

Positive expression of p40 was observed in cuboidal tumor cells (Figure 2) and basal cells of glandular structures of bronchi (Figure 3) in all 18 cases of SHL (18/18, 100%), No positive expression of p40 could be found in polygonal tumor cells (0/18, 0%) and normal alveolar epithelium surrounding the tumor. In all 18 cases of SHL, TTF-1 (Figure 4) and EMA were expressed in both polygonal tumor cells and cuboidal tumor cells(18/18, 100%). Cuboidal tumor cells showed cytoplasmic positivity for EMA and polygonal tumor cells displayed the main immuoreaction in membrane. CKpan was expressed in cuboidal tumor cells in some cases (14/18) but not in polygonal tumor cells in any case (0/18, 0%).Vimentin was diffusely expressed in polygonal tumor cells(18/18, 100%)and in cuboidal tumor cells in some cases(6/18). SPB positive expression was observed in cuboidal tumor cells (18/18, 100%), but not in polygonal tumor cells(0/18, 0%). Focal CgA and Syn positive expression in polygonal tumor cells was observed in only one case. CD34 was expressed only in the vascular endothelial cells. Positive expression of Ki-67 was scattered in a small number of tumor cells (less than 5% in all cases). SMA expression was negative in all tumor cells (Table 2, Table 3).

## Discussion

By studying SHL pathological manifestation and immunohistochemistry, it was demonstrated that there are two types of tumor cells in SHL: cuboidal tumor cells and polygonal tumor cells. Until now, the histogenesis of polygonal cells and cuboidal cells in SHL has remained unclear. Several theories on the tissue source of SHL have been proposed in the literature as follows: 1.Endothelialcell origin: in 1956, Liebow and Hubbell^1^ firstly reported seven cases of SHL. They proposed that SHL was a primary proliferation of blood vessels accompanied by secondary epithelial proliferation. It was suggested that SHL originated from vascular endothelial cells, and that its morphology was similar to that of skin sclerosing hemangioma. However, the endothelial origin was not supported by subsequent studies; only tumor vascular endothelial cells were positively labeled, while endothelial cell markers of both polygonal tumor cells and cuboid tumor cells were unlabeled^5^.Therefore, endothelial cells might simply be the squeezed alveolar epithelial cells. 2.Mesothelial cell origin, which was described by Katzensteinal, et al.^7^ The authors used electron microscopy data to support their belief that SHL originated from mesothelial cells. However, no morphological manifestation of mesothelial cells was found in subsequent electron microscopy studies. In addition, neither polygonal tumor cells nor cuboidal tumor cells expressed mesothelial cell-specific markers, such as CR and CK5/6, by the immunohistochemistry method. Therefore, this theory can be discarded, based on morphological observation and immunohistochemistry data. 3.Neuroendocrine cells origin: Xu et al.^8^ suggested that SHL originated from neuroendocrine cells. However later studies showed that in some cases a small amount of polygonal tumor cells expressed neuroendocrine markers. In our study, expression of CgA and Syn in polygonal tumor cells was observed in only one case, and no electron dense granules of typical neuroendocrine particle morphology were observed under the electronmicroscope. We have not found sufficient evidence to support SHLneuroendocrine origin, because the small amount of CgA and Synpositive cells might be the result of differentiation of tumor cells towards neuroendocrine cells. 4.Primitive alveolar epithelial cellorigin^3^,^4^,^5^,^6^:Through the immunohistochemical study of 100 SHL cases, Devouassoux et al.^3^ found that TTF-1, SPB, EMA and CK positive expression rates in tumor cells were very high, and that TTF-1 and SPB were the characteristic antigens of alveolar epithelial cells. This strongly suggested that SHL originated from the primitive respiratory epithelium. Hill and Kennedy^5^,^9^ proved that SHL tumor cells had the characteristics of alveolar cells by electron microscopy. Therefore, they believed that SHL might originate from immature alveolar epithelial cells, and could be differentiated to alveolar cells,Clara cells and neuroendocrine cells. The results of the present study proved that the cuboidal tumor cells expressed CKpan, SPB, TTF-1 and EMA, and polygonal cell tumor cells expressed TTF-1 and EMA, which supports the SHL primitive alveolar epithelial cell origin. The lack of reaction of Syn, CgA,CD34, and SMA supported the pulmonary epithelium origin, rather than neuroendocrine or vascular-endothelium. In addition, we found that p40 was exclusively expressed in cuboidal tumor cells,not in polygonal tumor cells and alveolar epithelial cells in tumor-surrounding normal lungtissue. p40 antibody(antibody recognizing ΔNp63 only)is a p63 isoform. The p63 gene encodes diverse mRNA isoforms,which are generated by the activity of two different promoters, one of which is internal to exon 3, which lead to the accumulation of TAp63 isoforms acting as TA agents, favoring cell cycle arrest with apoptosis and cell differentiation induction and ΔNp63-p40 isoforms,acting as negative dominant agents, which stimulate cell proliferation and block apoptosis with unrestrained tumor cell growth. In particular, recent data has indicated important roles in adult stem cell and cancer stem cell regulation^11^. p63 was consistently expressed in basal cells of normal epithelia, such as skin,urothelium, ectocervix and vagina, and basal cells of glandular structures of bronchi, and prostate. Importantly, these cells predominantly expressed the ΔNp63 isotype at about 100-fold to TAp63^12^. ΔNp63 was thought to function as a stem cell factor, responsible for maintaining cells in an uncommitted state with regenerative potential–a role that may be recapitulated in tumors derived from these cells^12^,^13^. Elevated expression of ΔNP63 isoforms has been found in several carcinomas, indicating an oncogenic role of ΔNP63 isoforms^14^. According to Nobre et al.^15^, the single expression of ΔNp63 was seen in the stem-like cell populations and may contribute significantly to biological processes such as proliferation and differentiation of epithelia. William^16^ found that ΔNp63 was an oncogene that cooperates with Ras to promote tumor-initiating stem-like proliferation in vivo. Celeste^17^ found that ΔNp63 plays a fundamental role in early epidermal development and ΔNp63 was an anti-apoptotic Factor during epidermal development in Xenopus. All this suggests that the p40-positive cells may be undifferentiated pluripotent stem cells. Therefore, we hypothesized that p40-positive expressing cuboidal tumor cells might be the original alveolar epithelial cells in SHL, whereas polygonal tumor cells were differentiation manifestations of cuboidal tumor cell.

## Methods

The study was approved by the Institutional Ethics Committee of HuaianFirst People's hospital. All activities involving human subjects were done under full compliance with government policies and the Helsinki Declaration. Written informed consent was obtained from all study subjects. All SHL cases were diagnosed with surgical resection and histopathological examination in our hospital. Clinical data were reviewed from the patients' charts. All specimens were fixed by 10%neutral formalin, embedded in paraffin and the slices were approximately 4 μm, Hematoxylin-eosin stained, and diagnosis of SHL was confirmed by two doctors with the result of light microscopy. Further immunohistochemical staining was performed on paraffin sections labeled by the EnVision method. Antigen retrieval was effected by pressure cooking in EDTA buffer, pH 8.0 for 2.5 minutes^2^. Primary antibodies were anti-p40 (5–17, CalBiochem/END Bioscrience, cat, NO, PC373), anti-Cytokeratinpan (CKpan, AE1/AE3, DaKo), anti-Epithelical Membrane Antigen (EMA,V9, DaKo), anti-Surfactant Protein B (SPB, AB3780, Chemicon International), anti-vimentin (Vim384, DaKo),anti-Thyroid Transcription Factor-1 (TTF-1, 8G7G3/1, DaKo,) anti-CD34(QBEnd/10, DaKo), anti-Ki67 Antigen (M1B5, DaKo), anti-Synaptophysin (Syn, SY38, DaKo, anti- Chromogranin A (CgA, DAK-A3, DaKo), and anti- Smooth Muscle Actin (SMA, 1A4, DaKo). The reaction was visualized by using DAB and then the sections were counter stained with hematoxylin. Cuboidal tumor cells and polygonal tumor cells were separately counted. For p40, TTF-1 and Ki-67staining, yellowish-brown granule in nuclei were considered as positive; for CKpan, EMA, vimentin, SPB, CgA, Syn CD34 and SMA staining, yellow or brown granules in a membranous or cytoplasmic pattern were considered as positive. For all antibodies, the percentage and positive intensity was calculated based on the actual number of positive cells; Immunoreactivity was rated semiquantitatively on a scale from negative (−) to positive (+). The number of counted cells < 10% was considered negative (−); the number of counted cells > 10%was positive (+)^2^,^3^. Staining intensity: “+” indicated weak,++ moderate of strong. The positive control was the positive reaction for each antibody; and negative control was the reaction in which primary antibody was replaced with PBS.

## Author Contributions

J.W., C.Z. and H.G.Q. wrote the main manuscript text and H.G.Q. prepared figures 1–4. All authors reviewed the manuscript.

## Figures and Tables

**Table 1:** Patient in SHL informations

N	Sex	Age	Course of disease(m)	Size of tumor (cm)	location of tumor
1	F	26	20 days	1	right lower lobes
2	F	58	24	4	right lower lobes
3	F	42	4	2	right lower lobes
4	F	37	3	2.5	right lower lobes
5	F	41	6	3.8	right lower lobes
6	F	40	1	3.2	right lower lobes
7	F	50	2	3.6	right lower lobes
8	F	33	1	2.8	right lower lobes
9	F	38	2	2.2	right lower lobes
10	F	44	3	3.3	left lower lobes
11	F	37	1	1.9	left lower lobes
12	F	45	1	2.7	left lower lobes
13	F	38	3	2.6	left lower lobes
14	F	49	1	3.1	right upper lobes
15	F	48	1	2.6	left upper lobes
16	F	44	1	3.8	left upper lobes
17	M	50	6	3.6	left upper lobes
18	M	51	10	3.6	right lower lobes

M: Male, F: Female,m: month, cm: centimetre.

**Table 2:** Expression of immunophenotype of P40,TTF-1,EMA,CKpan,Vimetin,SPB,CD34, SMA in 18 cases of SHL

Type of tumor cells	Positive counting cells															
	P40		TTF-1		EMA		CKpan		Vimetin		SPB		CD34		SMA	
	−	+	−	+	−	+	−	+	−	+	−	+	−	+	−	+
Cuboidal tumor cells	0	18	0	18	0	18	4	14	12	6	0	18	18	0	18	0
Polygonal tumor cells	18	0	0	18	0	18	18	0	0	18	18	0	18	0	18	0

Positive counting cells: +,the number of positive cells > 10%, −, the number of positive cells < 10%.

**Table 3:** Expression of immunostaining intensity of P40,TTF-1,EMA, CKpan,Vimentin, SPB in 18 cases of SHL

Type of tumor cells	Immunostaining staining intensity											
	P40		TTF-1		EMA		CKpan		Vimetin		SPB	
	+	++	+	++	+	++	+	++	+	++	+	++
Cuboidal tumor cells	0	18	0	18	18	0	14	0	6	0	18	0
Polygonal tumor cells	0	0	0	18	18	0	0	0	0	18	0	0

Immunostaining staining intensity:+ weak,++ moderate or strong.

## References

[b1] Liebow A. A. & Hubbell D. S. Sclerosing hemangioma (histocytoma,xanthoma) of the lung. Cancer. 9, 53–75 (1956). 13284701 10.1002/1097-0142(195601/02)9:1<53::aid-cncr2820090104>3.0.co;2-u

[b2] Chan A. C. & Chan J. K. Pulmonary sclerosing hemangioma consistently expresses thyroid transcription factor-1 (TTF-1): a new clue to its histogenesis. Am J Surg Pathol. 24, 1531–1536 (2000). 11075855 10.1097/00000478-200011000-00009

[b3] Devouassoux-Shisheboran M. *et al.* A clinicopathologic study of 100 cases of pulmonary sclerosing hemangioma with immunohistochemical studies: TTF-1 is expressed in both round and surface cells, suggesting and origin from primitive respiratory epithelium. Am J Surg Pathol 24, 906–916 (2000). 10895813 10.1097/00000478-200007000-00002

[b4] Yoo S. H. *et al.* Expression patterns of markers for type II pneumocytes in pulmonary sclerosing hemangiomas and fetal lung tissues. Arch Pathol Lab Med. 129, 915–919 (2005). 15974816 10.5858/2005-129-915-EPOMFT

[b5] Hill G. S. & Eggleston J. C. Electron microscopic study of so-called ‘pulmonary sclerosing hemangioma’: report of a case suggesting epithelial origin. Cancer. 30, 1092–106 (1972). 4342853 10.1002/1097-0142(197210)30:4<1092::aid-cncr2820300431>3.0.co;2-k

[b6] Illei P. B., Rosai J. & Klimstra D. S. Expression of thyroid transcription factor-1 and other markers in sclerosing hemangioma of the lung. Arch Pathol Lab Med. 125, 1335–1339 (2001). 11570910 10.5858/2001-125-1335-EOTTFA

[b7] Katzenstein A. L. *et al.* So-called sclerosing hemangioma of the lung, Evidence for mesothelial origin. Am J Surg Pathol. 7, 3–14 (1983). 6187231 10.1097/00000478-198301000-00001

[b8] Xu H. M. *et al.* Neuroendocrine dIfferendiation in 32 Cases of so called sclerosing hemangioma of the lung: identified by Immumuohistochemical and ultrastructural study. Am J Surg Patho1. 21, 1013–1022 (1997). 10.1097/00000478-199709000-00005 9298877

[b9] Kennedy A. Sclerosing haemangioma' of the lung: an alternative view of its development. J Clin Pathol. 26, 390 (1973). 4351732 10.1136/jcp.26.5.390-b PMC477761

[b10] Niho *et al.* Monoclonality of Both Pale Cells and Cuboidal Cells of Sclerosing Hemangioma of the Lung. American Journal of Pathology. 152, 1065–1069 (1998). 9546367 PMC1858231

[b11] Nekulova M. *et al.* The role of p63 in cancer, stem cells and cancer stem cells. Cell Mol Biol Lett. 16, 296–327 (2011). 21442444 10.2478/s11658-011-0009-9 PMC6275999

[b12] Yang A. *et al.* p63, a p53 homolog at 3q27–29, encodes multiple products with transactivating, death-inducing, and dominant-negative activities. Mol Cell. 2, 305–316 (1998). 9774969 10.1016/s1097-2765(00)80275-0

[b13] Senoo M. *et al.* p63 is essential for the proliferative potential of stem cells in stratified epithelia. Cell. 129, 523–536 (2007). 17482546 10.1016/j.cell.2007.02.045

[b14] Crook T., Nicholls J. M., Brooks L., O'Nions J. & Allday M. J. High level expression of delta- N-p63: a mechanism for the inactivation of p53 in undifferentiated nasopharyngeal carcinoma (NPC)? Oncogene. 19, 3439–3444 (2000). 10918601 10.1038/sj.onc.1203656

[b15] Nobre A. R., Albergaria A. & Schmitt F. p40: A p63 Isoform Useful for Lung Cancer Diagnosis–A Review of the Physiological and Pathological Role of p63. Acta Cytologica. 57, 1–8 (2013). 23221041 10.1159/000345245

[b16] William M. *et al.* ΔNp63α Is an Oncogene that Targets Chromatin Remodeler Lsh to Drive Skin Stem Cell Proliferation and Tumorigenesis. Cell Stem Cell. 8, 164–176 (2011). 21295273 10.1016/j.stem.2010.12.009 PMC4373450

[b17] Celeste T. *et al.* ΔNp63 is regulated by BMP4 signaling and is required for early epidermal development in Xenopus. Developmental Dynamics. 241, 257–269 (2012). 22170861 10.1002/dvdy.23706

